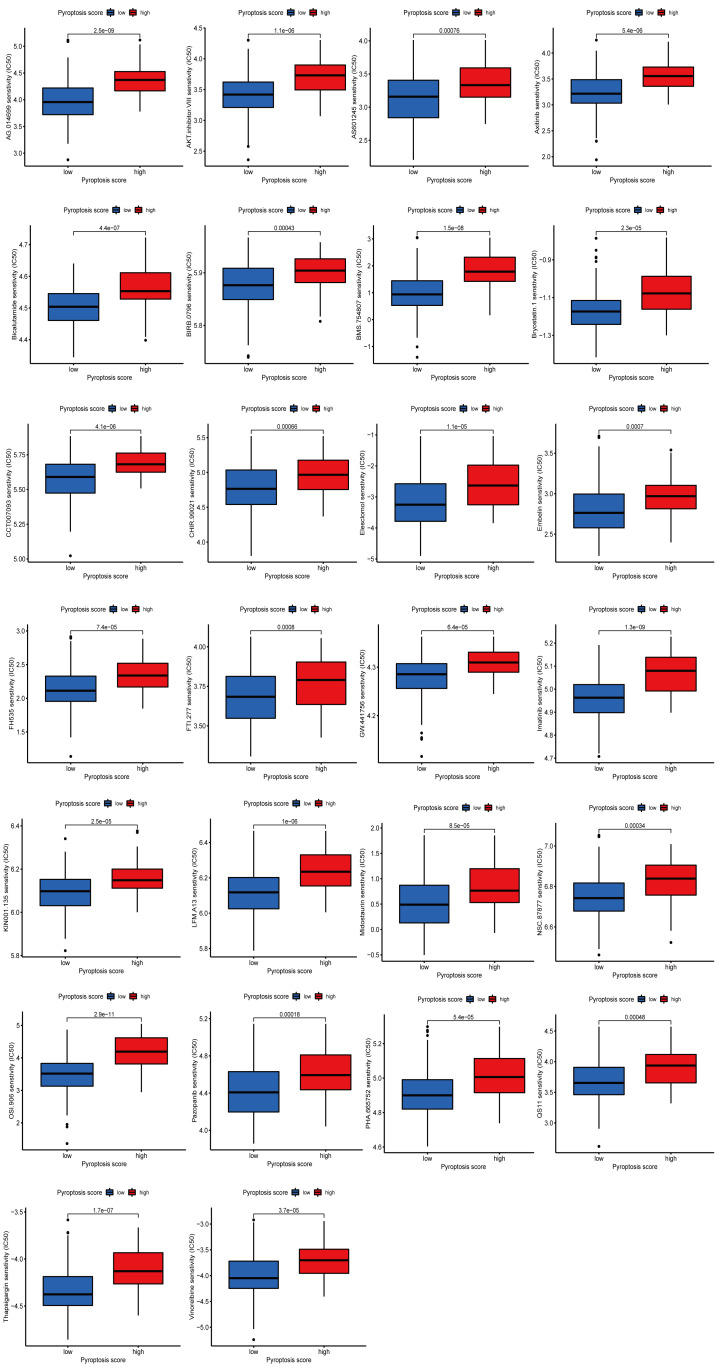# Correlations between the modification patterns mediated by pyroptosis-related genes, tumor microenvironment, and immunotherapy in soft tissue sarcoma

**DOI:** 10.1097/MD.0000000000038173

**Published:** 2024-05-17

**Authors:** Yang Cai, Jinzhi Meng, Yue Qiu, Xing Huang, Huawei Du, Jun Yao

**Affiliations:** a Bone and Joint Surgery, The First Affiliated Hospital of Guangxi Medical University, Nanning, People’s Republic of China.

**Keywords:** immunotherapy, prognosis, pyroptosis-related genes, soft tissue sarcoma, tumor microenvironment

## Abstract

Soft tissue sarcoma (STS) incidence, progression, and metastasis are tightly linked to the tumor microenvironment (TME). The modification patterns mediated by pyroptosis-related genes (PRGs) in STS are unknown regarding the immune cell infiltration landscape of TME, immunotherapy effect, and prognostic value. First, we downloaded STS samples from the Cancer Genome Atlas (TCGA) and gene-expression omnibus (GEO) databases. Based on 52 PRGs, 2 pyroptosis modification patterns were analyzed, and the associations of pyroptosis modification patterns with immune cell infiltration in the TME were elucidated systematically. To quantify PRG modification patterns in STS patients, we generated a pyroptosis scoring system using principal component analysis (PCA). We identified 2 distinct pyroptosis modification patterns in STS. Compared to PRG cluster A, the prognosis of cluster B was better. These 2 pyroptosis modification patterns corresponded to different characteristics of immune cell infiltration in the TME and biological behaviors. In the pyroptosis scoring system, a high pyroptosis score was connected to higher immune cell infiltration, stronger immune surveillance, immune-killing effects on tumor cells, and better clinical benefits. The results from 3 anti-PD1/PD-L1-treated immune cohorts demonstrated that higher pyroptosis scores are also closely connected to better immunotherapy results. We demonstrated that pyroptosis modification is essential to the STS microenvironment. Moreover, the pyroptosis score is a reliable and independent prognostic factor in STS patients, enabling a richer understanding of the STS microenvironment and the screening of immunotherapy candidates, predicting the immunotherapeutic effects for individual STS patients, and guiding the use of chemotherapy drugs.

## 1. Introduction

As a malignancy, soft tissue sarcoma (STS) originates from mesenchymal tissues, and more than 60 types of STS are identified so far.^[1]^ STS accounts for 1% to 2% of all cancers in humans and has a low incidence,^[2,3]^ possibly affecting 1 in 100,000 people.^[4]^ STS occurs in all parts of the body, most commonly in the limbs.^[5]^ STS have various causes, including radiation exposure and genetic factors,^[6–9]^ as well as exposure to carcinogenic chemicals.^[10]^ STS patients have a terrible prognosis, especially those with advanced sarcoma. Surgery combined with chemoradiotherapy is a major treatment option, and the prognosis of local lesions has improved greatly. However, recurrence is observed in half of the patients, and no effective or target therapeutic methods are available currently.^[11,12]^ The median overall survival (OS) of advanced patients is 1 to 2 years and is even <1 year for some patients.^[13]^ Given the STS treatment limitations, it is urgent to find a dependable prognostic indicator to guide therapy and improve the prognosis of STS patients.

Pyroptosis induced by CD8 + T cells is an immune-killing mechanism that induces inflammatory and programmed necrosis of tumor cells, closely related to the gasdermins protein family.^[14]^ Inducing the production and recreation of inflammatory cytokines, GSDMD and GSDME can cause a strong inflammatory response. At the same time, the cells become flattened and form scorched vesicles, eventually leading to pyroptosis.^[15–17]^ The tumor immune microenvironment (TIM) is significantly connected to the progression, metastasis, and therapeutic effect of tumors, as well as to pyroptosis. Additionally, significant positive associations of GSDMD expression in the TIM with CD8 + T-cell number and activity have been observed.^[18]^ Moreover, through a positive feedback mechanism, the pyroptosis of tumor cells can be induced by NK and CD8 + T cells in the TIM.^[19]^ Nevertheless, tumor microenvironment (TME) immune cells also help tumor cells to achieve immune evasions, such as antigen-presenting dendritic cells and T-/B-lymphocytes.^[20]^ Besides, the close connection of pyroptosis-related genes (PRGs) to the prognosis of malignant tumor patients has also been demonstrated.^[21,22]^ However, there are few studies on prognosis-associated PRGs in STS, and the landscape of immune cell infiltration in the microenvironment of PRGs-modified STS remains unclear. STS is a complex and heterogeneous tumor. The discovery of genetic characteristics associated with predicting STS prognosis is extremely important to its treatment and therapy in clinics.

An in-depth analysis of the immunological characteristics of TME can provide us with various new ideas for immunotherapy. Tumor immunotherapy has become a research hotspot, and tumor immune escape has been recognized as a hallmark of tumorigenesis.^[23]^ The therapeutic method of exerting an anti-tumor effect by manipulating the immune system is very promising, especially the blocking treatment of 3 immune checkpoints: CTLA-4, PD-1, and PD-L1 has achieved great success in tumor treatment and therapy, such as cutaneous melanoma, non-small-cell lung cancer, clear cell cancer, and hematological malignancies.^[24]^ However, not every STS patient is sensitive to immunotherapy. The search for effective biomarkers and cluster analysis methods is crucial for screening candidate patients for immunotherapy as well as designing strategies for individualized treatment, which has a practical significative value of improving STS patients’ prognosis.

Comprehensively analyzing the characteristics of immune cell infiltration in TME in PRG modification patterns and exploring the correlation between PRGs and immune response and prognosis will help find new biomarkers, screen patients suitable for immunotherapy, and provide new targets and drug options for STS therapy. Herein, we downloaded the transcriptome data and clinical characteristics of 182 STS patients from gene-expression omnibus (GEO) and the Cancer Genome Atlas (TCGA) databases. We obtained 2 different PRG modification patterns by comprehensively analyzing the characteristics of PRG-mediated TME cell infiltration. Further, we established a pyroptosis scoring system to characterize the immune landscape of STS, derived individualized PRG modification patterns in STS patients, and determined its value in guiding immunotherapy and chemotherapy drugs for these patients.

## 2. Materials and methods

### 2.1. Data collection and processing

We downloaded the transcriptome RNA sequence (FPKM value), copy number variation (CNV), clinical information, and single nucleotide polymorphism (SNP) of 120 STS patient samples from the TCGA-SARC cohort (https://portal.gdc.cancer.gov/). The data of somatic mutation were obtained via TCGA for STS patients (https://portal.gdc.carnec.gov/repository). Since only one normal sample was in TCGA, the GTEx database (https://xena.ucsc.edu/) was used to collect the data from 396 normal soft tissue samples, then calibrated and integrated. We placed normal samples before tumor samples and used the package “limma” to transform the data into TPM values for subsequent differential analysis. Meanwhile, the data of GSE17674 was collected and merged with the sequences of the transcriptome RNA from the TCGA-SARC cohort for further analysis using “limma” and “sva” R packages. Additionally, we downloaded the GSE17618 dataset, including information on 55 samples from the GEO database, to validate the OS calculated using the pyroptosis score.

### 2.2. Unsupervised clustering of PRGs

Based on previous studies, we identified 52 PRGs.^[25–27]^ Wilcox tests were used to assess the expression differences of PRGs between normal soft tissue and STS, and *P* < .05 was used as the screening criterion. Using the package “ConsensusClusterPlus,” we carried out the cluster analysis based on 52 PRGs expressions in STS patients.^[28]^ The steps were repeated 1000 times to ensure the reliability and stability of the clustering, and different PRG molecular subclusters of STS patients were obtained.

### 2.3. Single sample gene set enrichment analysis (ssGSEA) and gene set variation analysis (GSVA) between 2 PRGs molecular subclusters

For evaluating immune cell content in TME, the relative abundance difference of infiltrated immune cells was obtained using the ssGSEA method in the 2 PRG molecular subclusters of STS patients with “immune.gmt” and “GSEABase” R packages. To evaluate the genes in the combined cohorts of TCGA-SARC and GSE17674 and explore genes-regulated pathways and functions, we employed the “GAVA” R package.^[29]^ Through the GSVA algorithm (*P* < .05), we obtained the main enrichment functional pathway of 2 PRG molecular subclusters (gene set: c2. cp. kegg. v7.4. symbols. Gmt).

### 2.4. Screening and enrichment analysis of differentially expressed genes (DEGs) among different PRG molecular subclusters

Under the criteria of adjusted-*P* < .05, 2 PRG molecular subclusters were screened using the package “limma,” pyroptosis-associated DEGs were obtained, and the Gene ontology (GO) and Kyoto Encyclopedia of Genes and Genomes (KEGG) pathway analyses were conducted using the “ClusterProfiler” R package.^[30,31]^ For labeling and visualizing DEG functions, molecular functions (MF), biological processes (BP), and cellular components (CC) were applied.

### 2.5. Unsupervised clustering analysis of genes related to pyroptosis prognosis

Univariate Cox regression was carried out on DEGs using the package “survival” to obtain the prognosis-associated DEGs in STS patients. Then, through consistent unsupervised clustering analysis, patients were separated into clusters A and B, and the OS curves were drawn using the packages “survival” and “survminer.” At the same time, we also constructed a heatmap of prognostic genes in different gene clusters.

### 2.6. Establishment of a pyroptosis scoring system

We obtained several pyroptosis modification patterns in STS from the previous analysis step, but these patterns did not give a quantitative index for each individual. Thus, we constructed a pyroptosis scoring system to quantitatively evaluate the modification pattern of pyroptosis genes in each STS patient. According to the genes associated with prognosis, we conducted principal component analysis (PCA) and extracted the principal components 1 and 2 (PC1 and PC2) as feature scores.^[32]^ This method focuses on the scores of the set of the largest and highly correlated (or negatively correlated) gene blocks while eliminating the scores of genes that do not track other members as much as possible. The positive and negative associations of the pyroptosis gene signature with PC1 and PC2 were determined. Then the score of pyroptosis was obtained by formula:


$$\begin{array}{l} Pyroptosis\;score=\sum PC1_{i}+\sum PC2_{j} \end{array}$$

Where “i” and “j” indicate gene-expression associated with pyroptosis phenotype. According to the maximum selection rank statistics, we got a high pyroptosis score group (HPSG) and a low pyroptosis score group (LPSG) and used them for subsequent analysis.

### 2.7. Correlation analysis of pyroptosis score with genomic mutations

To evaluate the somatic mutations in HPSG and LPSG, the “maftools” R package was applied, and the top 20 most frequently mutated genes were obtained, as well as the characteristics and types of these mutations. According to the optimal cutoff value, we split STS patients into high and low tumor mutation burden (TMB) groups and analyzed their OS differences by combining TMB with pyroptosis scores.

### 2.8. Analysis of the correlation between immunotherapy and pyroptosis score

Immunotherapy for STS has been a research hotspot because it can improve the prognosis of patients.^[33]^ Unexpected results have been achieved with immune checkpoint blocking (ICB) therapies based on PD-L1, PD-1, and CTLA-4. Thus, we analyzed the difference between the 3 ICB genes in the HPSG and LPSG using the “limma” R package. We downloaded 3 cohorts for verification to evaluate the pyroptosis score values in immunotherapy. The detailed data of the atezolizumab (anti-PD1 antibody)-treated advanced urothelial carcinoma (IMvigor210 cohort) were obtained from http://research-pub.Gene.com/imvigor210corebiologies/. The detailed data and clinical information of GSE93157 and GSE78220 were obtained from GEO. Validation queues were converted to TPM values through the “limma” R package.

### 2.9. Analysis of drug sensitivity

To explore the differences in chemosensitivity between HPSG and LPSG, the half maximal inhibitory concentration (IC_50_) value of commonly used drugs for chemotherapy in STS therapy was calculated utilizing the “pRRophetic” R package. The rank sum test was used, and a *P* < .001 was set as the filter condition.

### 2.10. Statistical analysis

For analyzing and processing the data, Perl (v. 5.3.0) and R (x64 v. 4.1.3) were carried out. A *P* < .05 was set as a significant difference. The “surv cutpoint” function was used to test each potential cutpoint repeatedly to determine the greatest rank statistic and separate patients into HPSG and LPSG. The “survminer” R package was used for survival analysis. To compare 2 or more groups and 2 groups, the Kruskal-Wallis and the Wilcox tests were employed respectively. Multivariate Cox regression was used to evaluate the prognostic value of the pyroptosis score. The difference in survival was compared using Log-rank tests and Kaplan–Meier (KM) curves.

## 3. Results

### 3.1. Genetic variation landscape of PRGs between STS and normal tissues

**Figure 1.**
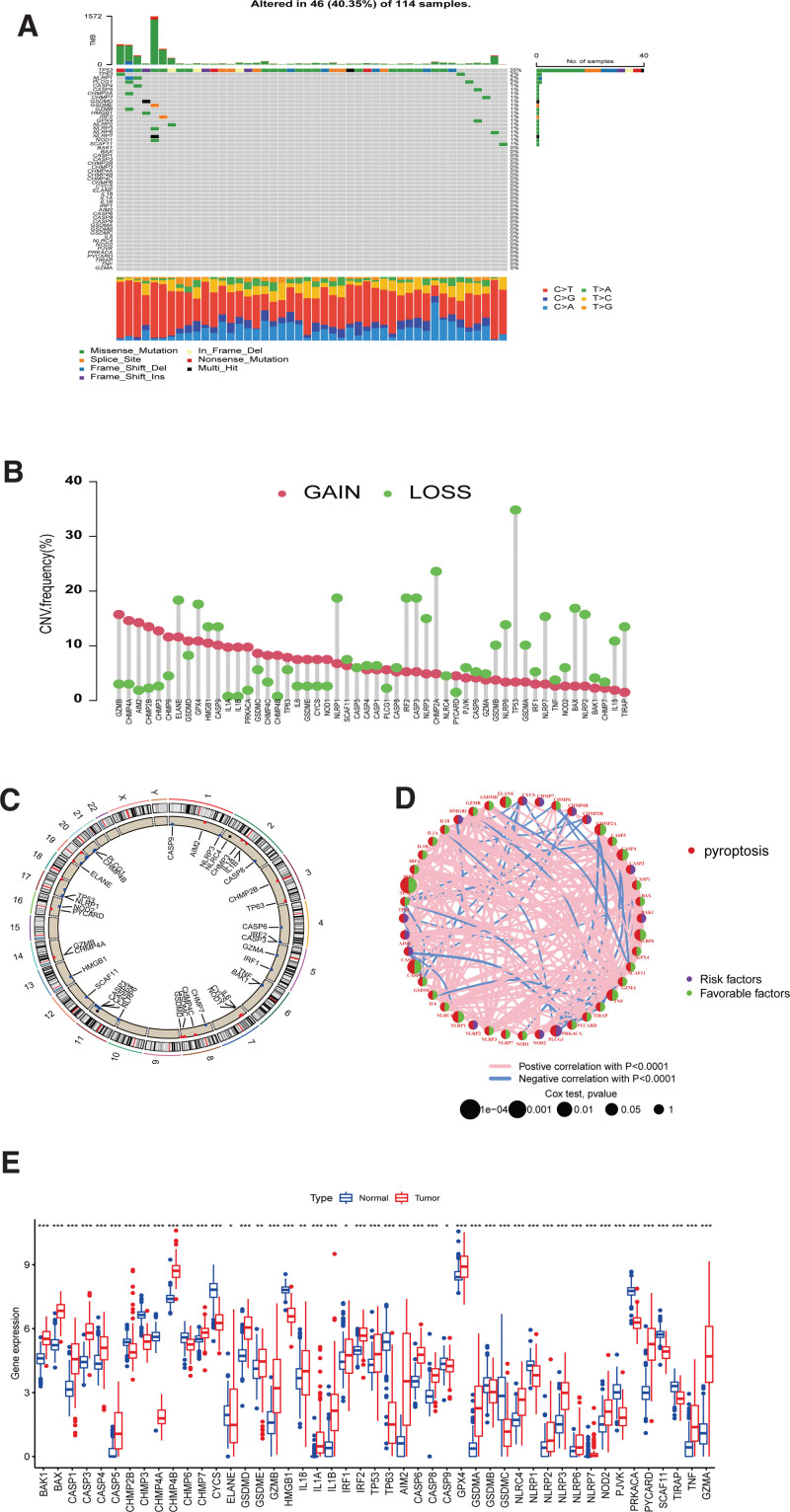
PRGs genetic landscape and expression in STS. (A) Frequencies of PRGs mutation in TCGA-SARC. (B) CNV frequency diagram of 52 PRGs in TCGA-SARC, Red: copy number increases. Green: copy number decreases. (C) CNV locations of PRGs on 23 chromosomes in TCGA-SARC. (D) Prognostic network diagram of PRGs. Left semicircles: PRGs. Right semicircles: the risk of the genes. Purple: high-risk genes. Green: low-risk genes. The larger the circle, the more likely the gene is to be prognostic-related. Regarding the line between 2 genes, a positive correlation is indicated by red, and blue represents the opposite. (E) Expression of the 52 PRGs STS and normal soft tissues. Normal soft tissue (blue) and STS (red). *, **, *** represent the *P* values <.05, .01, and .001, respectively. CNV = copy number variation, PRGs = pyroptosis-related genes, STS = soft tissue sarcoma, TCGA = The Cancer Genome Atlas.

In TCGA-SARC cohort, the CNV and somatic mutation of 52 STS PRGs were investigated. We found mutations in 46 of 114 samples (40.35%), and the top 4 mutations were TP53 (35%), TP63 (2%), NLRP1 (2%), and PLCG1 (2%) (Fig. 1A). The 52 PRGs prevalently had somatic cell CNV, which significantly increased in GZMB, CHMP4A, and AIM2 and significantly decreased in TP53, CHMP2A, and CASP3 (Fig. 1B). The CNV circle diagram displays the position of each PRG on the chromosome (Fig. 1C). In the prognostic network of PRGs in STS patients, we found that IRF2, ELANE, and NLRP6 were the most significantly associated with prognosis (Fig. 1D). Moreover, 31 survival- and prognosis-related PRGs were obtained using univariate Cox survival analysis and KM curves. The prognostic survival curves for these PRGs are shown in Supplementary Figure 1, http://links.lww.com/MD/M581. Then, RNA sequences of TCGA-SARC cohort samples and 396 normal soft tissue from the UCSC Xena platform were pooled to assess the expression differences of PRGs between normal soft tissue and STS (Fig. 1E) to help understand whether genetic variation plays a role in STS. A higher level of PRGs in some CNV gain was observed in STS than in normal soft tissue samples, including GZMB and AIM2, while there was a reduction in PRGs expression of some CNV loss, including TIRAP and NLRP1. These results suggested that CNV might be involved in the regulation of PRGs expression and the potential role of imbalanced PRG expression for STS.

### 3.2. Consensus cluster analysis of PRGs in STS

**Figure 2.**
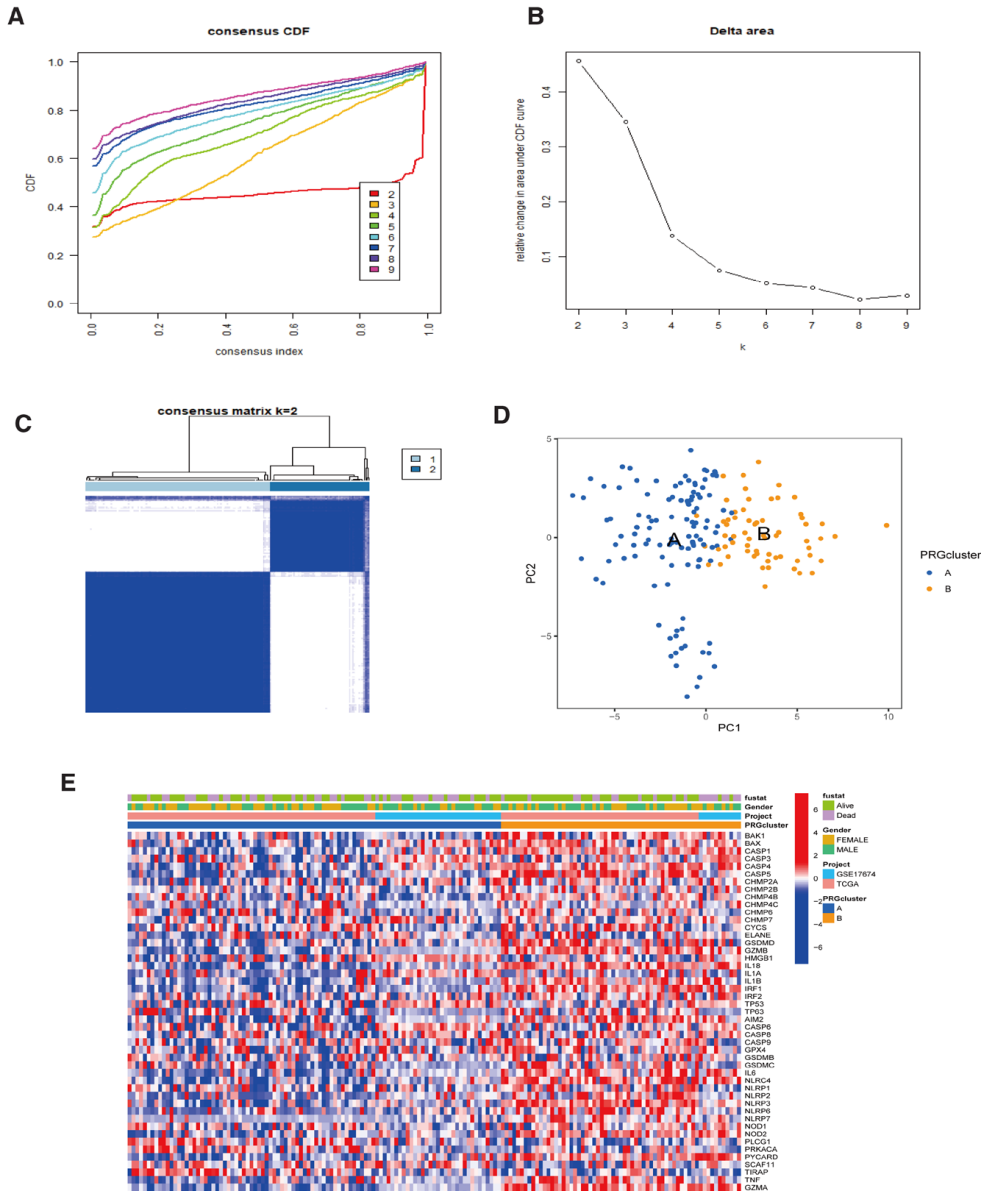
Cluster analysis of PRGs expression based on STS. (A, B) Relative changes in the area under the CDF curve for consistent clustering for K = 2–9. (C) Consensus clustering matrix for K = 2. (D) PCA of 2 PRG modification patterns. (E) Unsupervised clustering heatmap and clinical features of PRGs in 2 PRG molecular subclusters. High (red) and low (blue) expressions. PCA = principal component analysis, PRGs = pyroptosis-related genes, STS = soft tissue sarcoma.

Using the “ConsensusClusterPlus” R package, we clustered 52 PRGs unsupervised to better understand their expression characteristics and function in STS. First, we removed the sample information of normal soft tissues. Then, according to the empirical cumulative distribution function (CDF) diagram, we found that K = 2 was the best choice (Fig. 2A and B) and divided STS patients into PRG clusters A and B (Fig. 2C). Furthermore, we used PCA in the 2 PRG clusters and found that pyroptosis expression profiles differed significantly (Fig. 2D). Next, we obtained 2 PRG modification patterns and drew a PRG clustering heatmap, indicating that the expression of these 52 PRGs in cluster B was dramatically upregulated (Fig. 2E).

### 3.3. TME characteristics of different PRG molecular subclusters

**Figure 3.**
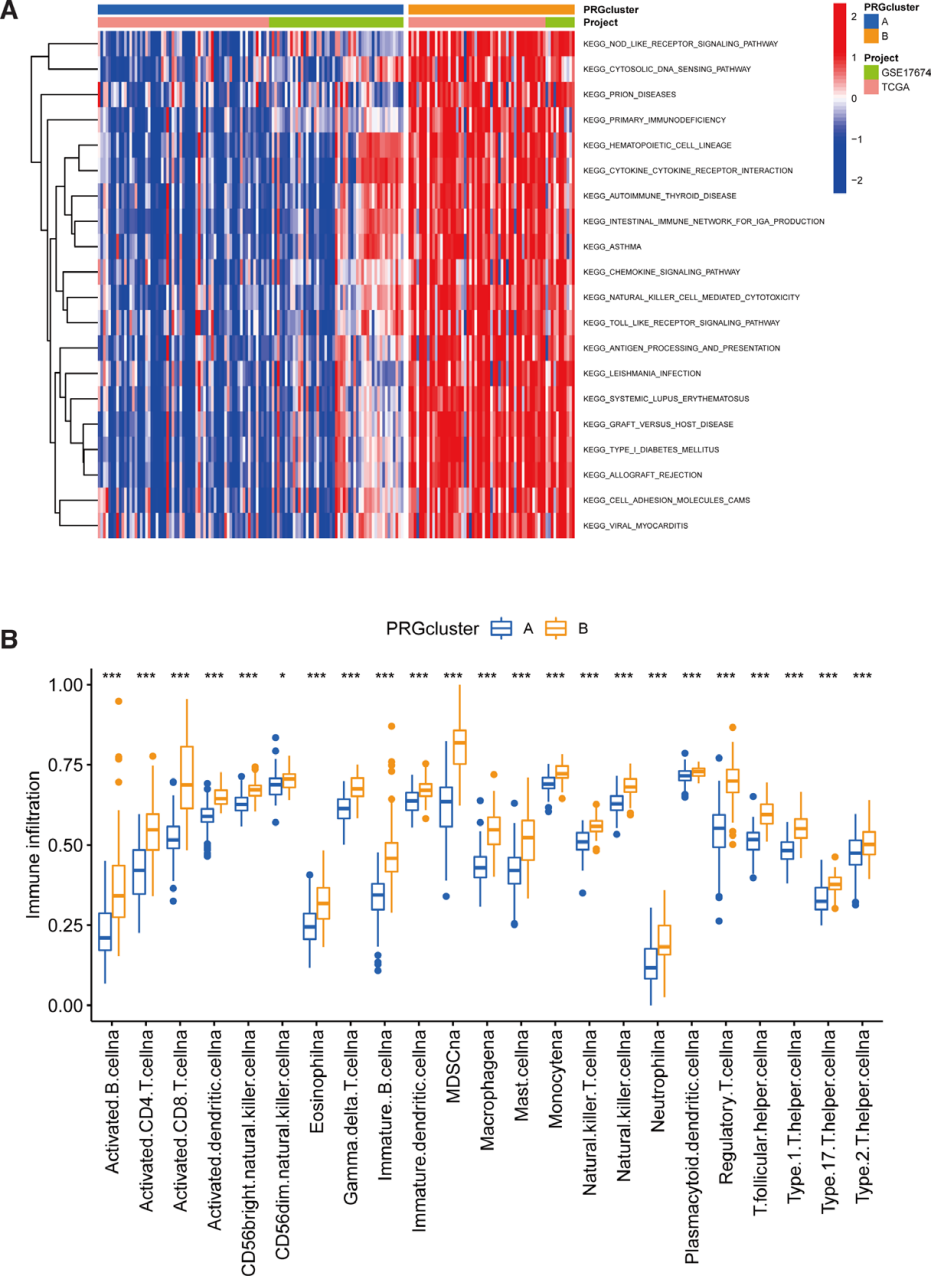
GSVA and ssGSEA for the 2 PRG modification patterns. (A) GSVA between PRG clusters A and B. Red: significant pathway enrichment. Blue: less pathway enrichment. (B) Infiltration of immune cells between 2 PRG subclusters in STS. Cluster A was showed as blue color, and cluster B was showed as yellow color. *, **, *** represent the *P* values <.05, .01, and .001, respectively. GSVA = gene set variation analysis, PRGs = pyroptosis-related genes, ssGSEA = single sample gene set enrichment analysis, STS = soft tissue sarcoma.

To further understand the biological functions of molecular subclusters of PRGs under different modification patterns, we conducted GSVA enrichment analysis. By comparing PRG clusters A and B, significant enrichments of cluster B in pathways involved in the activation of immune cells, such as Nod-/Toll-like receptor signal pathways, antigen processing and presentation, cytokine and chemokine mediated signal pathways, and natural killer cell-mediated cytotoxicity. However, the same pathways showed reduced enrichment in PRG cluster A (Fig. 3A). Next, we compared immune cell infiltration between the 2 PRG molecular subclusters using the ssGSEA. We found a significant difference in the abundance of immune cell infiltration between the 2 molecular subclusters. The infiltration abundance of all immune cells, such as activated CD4, CD8T, B cells, immune B cells, and activated dendritic cells, was significantly higher in the PRG cluster B (Fig. 3B). This result suggested that PRGs expression can guide the immunotherapy of STS patients.

### 3.4. Identification of different PRG molecular subclusters phenotype-associated genes

**Figure 4.**
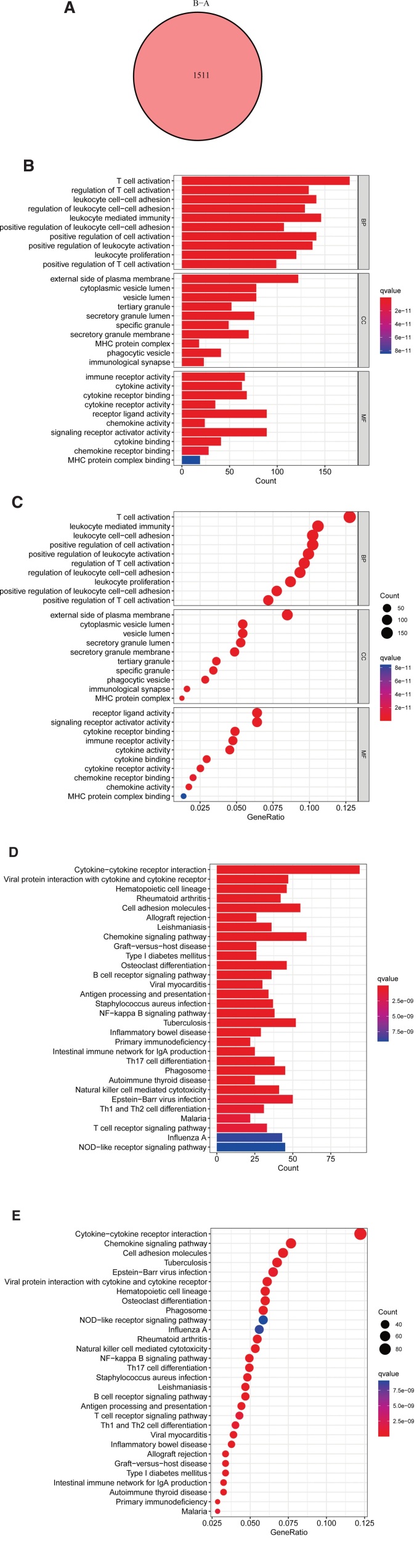
Identification and enrichment of DEGs in pyroptosis. (A) Representative Venn diagram showing the intersecting genes for the 2 PRG modification patterns. (B, C) The analysis of KEGG enrichment for DEGs. (D, E) The analysis of the GO function enrichment for DEGs. DEGs = differential expression genes, PRGs = pyroptosis-related genes.

To investigate the potential biological function of each subcluster, we analyzed the mechanism of changes in PRG expression between the 2 modification patterns. To screen the 1511 overlapping DEGs associated with PRG phenotypes, the “limma” R package was applied (Fig. 4A). To show the related action pathways of different DEGs, we utilized the “clusterProfiler” R package to perform GO and KEGG enrichment analysis on DEGs. GO is divided into 3 sections: BP, CC, and MF. In BP, DEGs were enriched in positive regulation of leukocyte activation, positive regulation of cell activation, leukocyte cell-cell adhesion, leukocyte-mediated immunity, and T-cell activation. In CC, they were mainly enriched in the vesicle lumen, cytoplasmic vesicle lumen, and the external side of the plasma membrane. MF was mainly enriched in immune receptor activity, receptor-ligand activity, cytokine receptor binding, and signaling receptor activator activity (Fig. 4B and C). In the KEGG analysis, enrichments of DEGs in cytokine-cytokine receptor interaction, cell adhesion molecules, and chemokine signaling pathway were observed (Fig. 4D and E). The above results surprisingly indicated that these genes were enriched in biological functions related to immunity, confirming that immune regulation of TME is profoundly influenced by PRG modification.

### 3.5. Identification of DEGs subclusters

**Figure 5.**
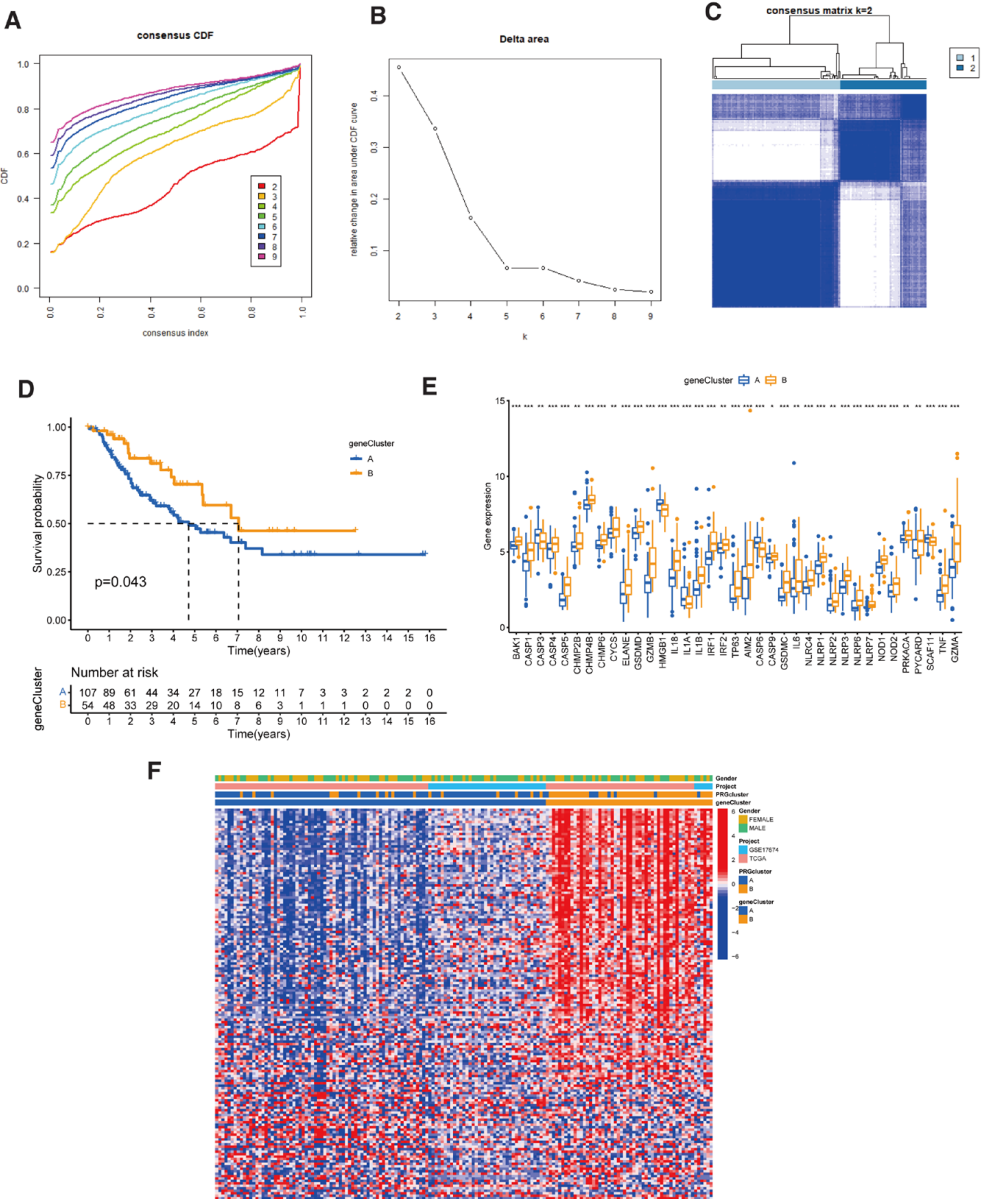
Consensus cluster analysis of genomic phenotypes based on DEGs. (A, B) CDF for consistent clustering for K = 2–9. (C) Gene consensus clustering subclusters for K = 2. (D) OS between 2 gene clusters. (E) Representative block diagram showing the differentially expression PRGs between 2 different gene clusters. (F) Heatmap of differential genes and clinical traits in 2 gene clusters. DEGs = differential expression genes, PRGs = pyroptosis-related genes.

Using the univariate Cox regression analysis, we screened 160 DEGs associated with prognosis from 1511 DEGs to further analyze the immune regulation mechanism of phenotype-related DEGs. Using unsupervised cluster analysis, we confirmed that the best choice was K = 2, and 2 clusters (A and B) were categorized (Fig. 5A–C). We plotted the KM survival curve (OS) between these 2 gene clusters, and the survival prognosis was better for cluster B (Fig. 5D). Differential expression analysis of PRGs between different gene clusters showed that gene cluster B exhibited significant overexpression of most of the 52 PRGs, and only a small number of PRGs were obviously expressed in cluster A, such as SCAF11, CASP6, and L1A (Fig. 5E). Furthermore, we drew a heatmap combining different clinical features, PRG molecular subclusters, and gene clusters. Interestingly, the expression of prognostic DEGs was profoundly upregulated in gene cluster B (Fig. 5F). Through the above analysis, we found that the expression of PRGs was closely related to the prognosis of STS patients. The higher the expression of PRGs, the better the patient prognosis, which is of great significance for guiding clinical treatment.

### 3.6. Generation of the prognostic PRG score signature

**Figure 6.**
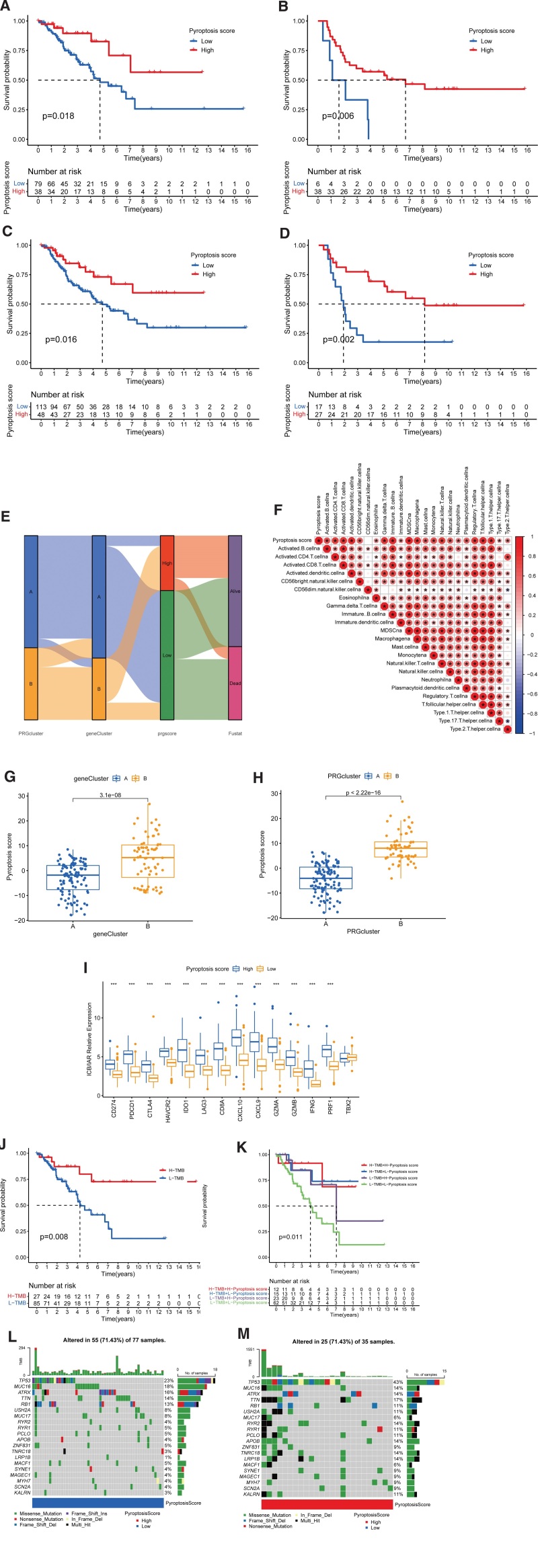
Establishment of the score of pyroptosis in STS. (A–D) Survival in HPSG and LPSG. Red: high expression. Blue: low expression; (A) TCGA. (B) GSE17674. (C) Combined cohort (TCGA and GSE17674). (D) Survival analysis curve of GSE17618 in HPSG and LPSG. (E) Sankey diagram of the distribution in survival status, pyroptosis scores, gene clusters, and PRG clusters. (F) Analysis of the immunologic correlation of pyroptosis score with immune cell infiltration. Red: positive correlations. Blue: negative correlations. * Indicates significant association between immune cells and pyroptosis score. (G, H) The differences in the scores of pyroptosis between the PRG modification patterns and 2 gene clusters (*P* < .001). (G) Gene clusters. (H) PRG clusters. (I) Differences in the expression of ICB (LAG3, IDO1, HAVCR2, CTLA4, PDCD1, and CD274) and IAR (GZMB, TBX2, CXCL9, PRF1, CXCL10, IFNG, CD8A, and GZMA) genes between HPSG and LPSG (* *P* < .05; ** *P* < .01; *** *P *< .001). (J) Survival of high and low TMB patients in TCGA-SARC (*P* < .001). (K) Kaplan–Meier survival curve classified by TMB groups-pyroptosis scores groups in TCGA-SARC (*P* < .001). (L, M) Waterfall diagram of tumor mutation genes between HPSG and LPSG. (L) LPSG. (M) HPSG. HPSG = high pyroptosis score group, LPSG = low pyroptosis score group, PRGs = pyroptosis-related genes, STS = soft tissue sarcoma, TCGA = The Cancer Genome Atlas, TMB = tumor mutation burden.

Using these prognostic-related DEGs, a scoring system of pyroptosis was established to quantitatively assess the patterns of PRG modification in each STS individual. In TCGA-SARC, GSE17674, and all STS patients’ cohorts, the survival analysis revealed that HPSG had a considerably better prognosis than LPSG (Fig. 6A–C). The same outcomes were attained for the dataset used for validation (GSE17618) (Fig. 6D). Sankey diagrams visually demonstrated that the PRG cluster B was significantly expressed in most STS patients. Gene cluster B was included in the HPSG and had a substantial survival prognostic benefit (Fig. 6E). To better display the characteristics of PRGs, a correlation analysis was carried out between pyroptosis score and TME immune cells. We found that compared to the LPSG, the HPSG showed a significant positive correlation with anti-tumor cells, such as type 1 and 17 T helper cells, Natural killer cells, activated dendritic cells, Natural killer T cells, Activated CD4+, CD8 + T cells, and activated B cells (Fig. 6F). Then, using the Kruskal-Wallis test, we performed a variation analysis in pyroptosis scores between PRG molecular subclusters and gene clusters. PRG cluster B and geneCluster B exhibited greater pyroptosis scores (Fig. 6G and H). The above results demonstrated that we could use pyroptosis scores to predict the characteristics of the infiltration of immune cells into TME and PRG modification patterns of STS in specific patient samples. After that, we predicted the immunotherapeutic response of STS patients by examining differences in the expression of 6 genes involved in immune checkpoint transcription, also known as ICB genes: LAG3, IDO1, HAVCR2, PDCD1/PD-1, and CTLA4, CD274/PD-L1, and 8 immune activation-related (IAR) genes between HPSG and LPSG (Fig. 6I).^[34–36]^ All genes, except TBX2, showed high expression in HPSG. This result implied that pyroptosis scores and immunotherapy responses are interconnected.

Based on the best cutoff value, we categorized TCGA-SARC cohort into low and high TMB groups and plotted KM survival curves. Compared to the low TMB group, the high TMB group exhibited a better survival prognosis (Fig. 6J). Combined analysis of the pyroptosis score groups and TMB groups revealed that the H-TMB + H-pyroptosis score group had the best survival prognosis, while the L-TMB + L-pyroptosis score group had the worst (Fig. 6K). At the same time, we plotted the tumor somatic mutation waterfall of the 10 highest mutant genes in the HPSG and LPSG (Fig. 6L and M). We found that the somatic mutation frequency was the same in HPSG and LPSG (71.43%) but a higher somatic mutation frequency in the HPSG for TP53 (55 vs 19%) and TTN (17 vs 14%). However, the opposite was observed in MUC16 and ATRX. These findings indicated that the pyroptosis score is unaffected by high or low TMB and can be used as an independent predictor.

### 3.7. Potential value of pyroptosis score in predicting response to immunotherapy and drug sensitivity analysis

**Figure 7.**
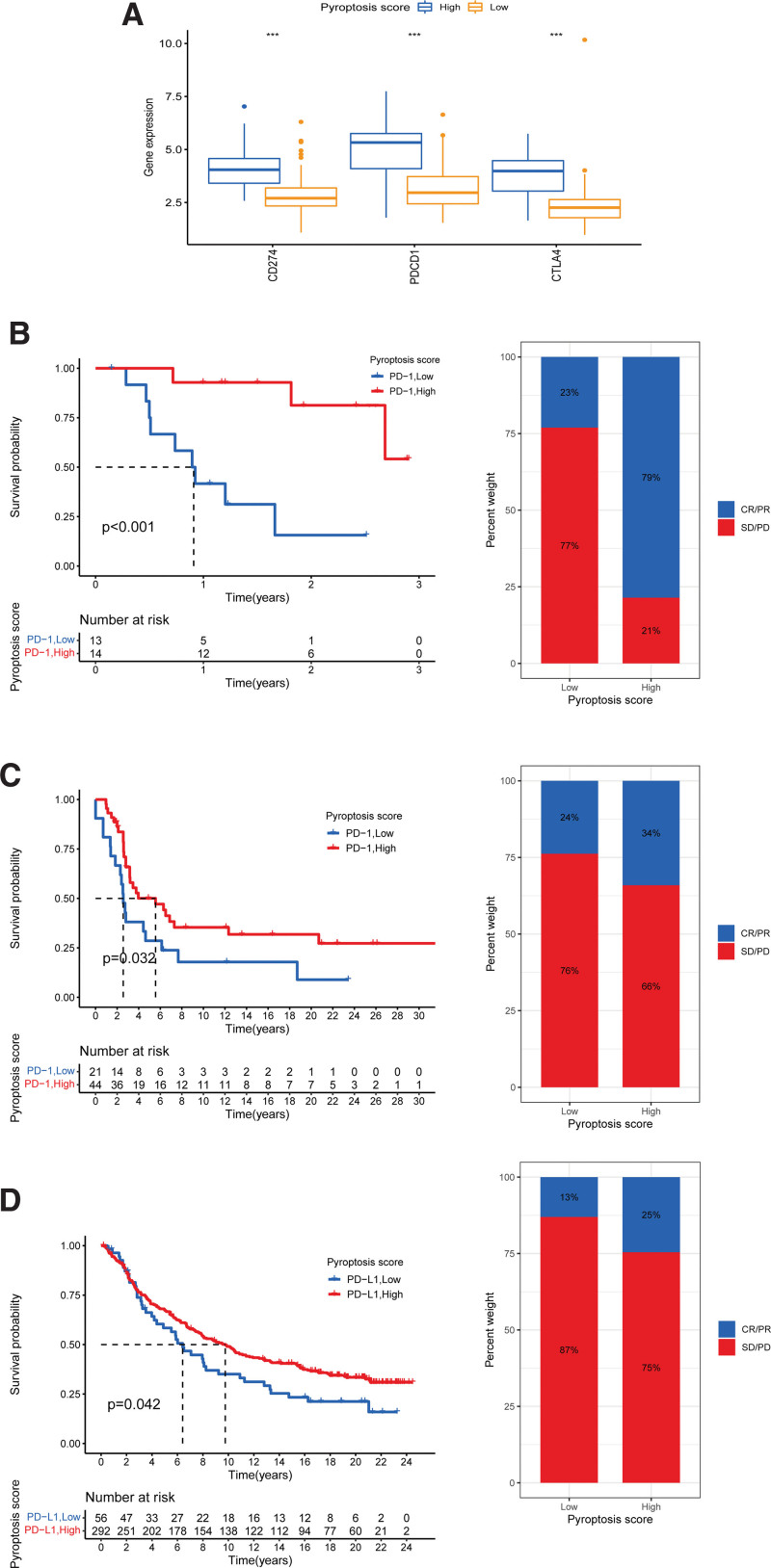
Role of pyroptosis score in predicting the efficacy of immunotherapy and drug sensitivity analysis. (A) ICB gene-expression differences between HPSG and LPSG (***P < .001; **P < .01). (B, C) Kaplan–Meier survival curves across HPSG and LPSG in the cohort treated with anti-PD-1 treatment and the response to immunotherapy in STS patients (PR partial response, PD: disease progression, SD stable disease, and CR: complete response). (B) GSE78220 cohort. (C) GSE93157 cohort. (D) Survival curves across HPSG and LPSG in the anti-PD-L1 antibody administrated IMvigor210 cohort and the efficiency of immunotherapy in STS patients. HPSG = high pyroptosis score group, LPSG = low pyroptosis score group, STS = soft tissue sarcoma.

To determine pyroptosis score predictive value for the response to immunotherapy, we performed correlation studies between pyroptosis scores and 3 ICB genes, CTLA-4, PD-1, and PD-L1. The 3 ICB genes in the HPSG had a more significant expression (Fig. 7A). Then, 3 cohorts were applied to verify the value of the pyroptosis score in predicting the therapeutic response to ICB. In the anti-PD-1/anti-PD-L1-treated cohorts (GSE78220, GSE93157, IMvigor210), STS patients in the HPSG had a significant survival prognosis, and the response rates in the HPSG and LPSG were 79% versus 23%, 34% versus 24%, 25% versus 13%, indicating that STS patients in the HPSG had better clinical benefits and were more suited to immunotherapy (Fig. 7B–D). The study of the correlation between pyroptosis score and clinical chemotherapy drugs showed that the IC_50_ values in the HPSG were significantly lower than those in the LPSG for 37 chemotherapeutic drugs, including velipanib, selumetinib, afatinib, methotrexate, and paclitaxel. We also obtained 31 chemotherapeutic drugs with lower IC_50_ values in the LPSG, including axitinib, imatinib, and linitinib. Detailed drug sensitivity analyses are provided in Supplementary Figure 2–3, http://links.lww.com/MD/M582, http://links.lww.com/MD/M583. Overall, these results provided some clues for clinical chemotherapy programs.

## 4. Discussion

The first discovery of pyroptosis was defending against pathogenic damage, which is involved in many inflammation-related diseases.^[37]^ However, increasing studies have suggested that pyroptosis is significantly connected to the inhibition or promotion of tumor cell growth,^[38,39]^ which is dramatically regulated by various cellular components, cytokines, and chemokines in the TME.^[20,40]^ STS have a wide variety of histological types, and its immune infiltration landscape of the TME is significantly heterogeneous, which is matched with the genomic STS changes.^[41]^ This suggests that the PRG expression characteristics and the immune infiltration landscape of the TME play an important role in the prognosis of STS patients, which can provide individualized treatment plans for immunotherapy.

To obtain the expression characteristics and biological value of PRGs in STS patients, we performed mutation, CNV, and expression correlation analysis of 52 PRGs in TCGA-SARC and found significant expression differences and heterogeneous genomic alterations. PRGs change in tandem with tumor occurrence and prognosis. STS is usually accompanied by highly repetitive mutations in the genes encoding TP53 and RB1, and their genomic diversity and regulatory diversity are strongly linked to the molecular subtypes associated with the prognosis in STS patients.^[9]^ Pyroptosis can occur in esophageal cancer cells with increasing NLRP3 expression.^[42]^ However, in some cases, PRGs might also play an immunosuppressive role and promote the survival of tumor cells, such as HMGB1.^[43]^ Among the 2 pyroptosis modification patterns, the expression of PRGs was significantly upregulated in PRG cluster B. These 2 pyroptosis modification patterns presented different biological functions and TEM immune cell infiltration characteristics. The GSVA enrichment revealed that PRG cluster B had a more significant enrichment of immune cell activation-related pathways, which corresponded to the immune-inflammatory phenotype. For example, the Toll-like receptor 4 (TLR4) signaling pathway is significantly associated with mediating pyroptosis development.^[44]^ At the same time, through ssGSEA, a dramatically high infiltration level of immune cells with anti-tumor effects, such as CD4 + T cells, CD8 + T natural killer T cells, and B cells, was found in cluster B. Thus, cluster B was considered a hot tumor.^[45,46]^ The immune-inflammatory phenotype has abundant T-cell infiltration and plays a better role in immune surveillance and immune killing of tumor cells.^[47]^ Furthermore, we carried out GO and KEGG enrichment analyses on DEGs. Surprisingly, we observed significant connections between these genes to immune processes, such as immune cell activation and ligand-receptor binding. For example, NOD-like receptors and Toll-like receptors bind to their corresponding ligands and play a vital role in pyroptosis.^[48]^ Similarly, compared to cluster A, a better prognosis was observed in cluster B, which again illustrates that PRG modification can distinguish different STS patients and formulate different treatment strategies. Good prognosis is closely related to the TIM, suggesting that different PRG modification patterns are involved in forming different TME landscapes.

To have a profound understanding of TME cell infiltration characteristics, we need an integrated, comprehensive assessment of PRG modification patterns. Hence, we devised a scoring system that considers the variability and complexity of TME in people. As expected, STS patients in the HPSG had a more significant survival benefit than those in the LPSG. At the same time, a significantly positive association of the pyroptosis score with the level of anti-tumor immune cell infiltration was discovered. T lymphocytes are the primary immune cells involved in killing tumors. Previous studies have shown that the higher the degree of B cell infiltration, the better the prognosis benefit,^[13,49]^ which was the strongest prognostic factor.^[50]^ Interestingly, PRG cluster B and PRG gene cluster B, characterized by immune-inflammatory phenotypes, also showed higher pyroptosis scores. We also observed a significant association between the characteristics of pyroptosis scores with immune checkpoints. In the HPSG, high expression of ICB genes, such as PD-L1, PD-1, and CTLA4, was observed. ICB gene is the target of immune checkpoint inhibitors. T lymphocytes are the main immune cells involved in killing tumors in the human body, and their immune activation is determined by costimulatory and inhibitory signals (immune checkpoints).^[51]^ Immunosuppressive blocking therapy activates the anti-tumor activity of immune cells such as T lymphocytes. Then it mediates the production of chemokines and transduction of interferon signaling in the TME and finally kills tumor cells, which is the direction of future tumor-targeted therapy.^[52–54]^ The combined analysis of pyroptosis score groups and TMB groups also showed that the H-TMB + H-pyroptosis score group had a better survival prognosis, indicating the association of TMB with the pyroptosis score. At the same time, the immune cell infiltration with high TMB was higher, which might maintain the sensitivity of cancer patients to immunotherapy using anti-PD-1/PD-L1 antibodies. Comprehensive analysis suggests that pyroptosis score is an independent factor and reliable for STS patient prognosis and can be used to select immunotherapy candidates, predict the effectiveness of immunotherapy and guide the use of chemotherapy drugs.

For the verification of the accuracy of the pyroptosis score in predicting the PD-1/PD-L1 treatment effect, we used 3 cohorts (GSE78220, GSE93157, and IMvigor 210). As expected, STS patients in the HPSG had a higher objective response rate and better prognosis. Qiu and Bae et al evaluated the expression of immune biomarkers in STS and found that increased CTLA4, PD-1, and LAG3 were connected to a good prognosis.^[55,56]^ This result also indicated that STS patients who respond well to immunotherapy have higher pyroptosis scores, higher levels of immune cell infiltration, and stronger immune surveillance and immune-killing effects on tumor cells, which can explain the results of better OS in STS patients with high pyroptosis scores. Therefore, due to the prognosis prediction and the guidance for the immunotherapy strategy and targeted therapy program, the pyroptosis score might significantly benefit STS patients. Meanwhile, in the HPSG, 37 chemotherapeutic drugs were obtained with IC_50_ considerably lower than the LPSG, which might help explore new drug combination patterns or new targeting agents. Chemotherapy drugs such as paclitaxel and cisplatin turn caspase-3-mediated apoptosis into pyroptosis and cause the specific cleavage and activation of GSDME, which eventually cause pyroptosis and effectively impair the metastasis and growth of tumor cells.^[57,58]^ Combination chemotherapy might have a longer prognostic survival time.^[59]^ Different immune responses and gene mutations can lead to these differences in sensitivity to chemotherapy drugs between the HPSG and the LPSG.^[15]^ In summary, our current findings provided new insights for screening immunotherapy candidates, identifying the immunophenotype, and facilitating personalized immunotherapy regimens for STS.

## 5. Conclusion

Herein, we elucidated the broad underlying mechanisms of pyroptosis modification patterns in the STS microenvironment. Pyroptosis modification is connected to the TME and infiltration of immune cells. Because the patterns of pyroptosis modification are complex and heterogeneous, we developed a pyroptosis scoring system for quantitative assessment of PRG modification patterns in each STS patient, enhancing our understanding of cellular infiltration features in the TME and might aid in the identification of immunotherapy candidates, the development of tailored immunotherapy regimens, and the improvement of patient outcomes after immunotherapy.

## Acknowledgments

We thank all the team members for participating in this study.

## Author contributions

**Conceptualization:** Jun Yao.

**Data curation:** Yang Cai, Yue Qiu, Huawei Du.

**Formal analysis:** Huawei Du.

**Software:** Yang Cai.

**Supervision:** Jun Yao.

**Validation:** Xing Huang.

**Visualization:** Yue Qiu, Xing Huang.

**Writing – original draft:** Yang Cai.

**Writing – review & editing:** Yang Cai, Jinzhi Meng.

## Supplementary Material

**Figure:**
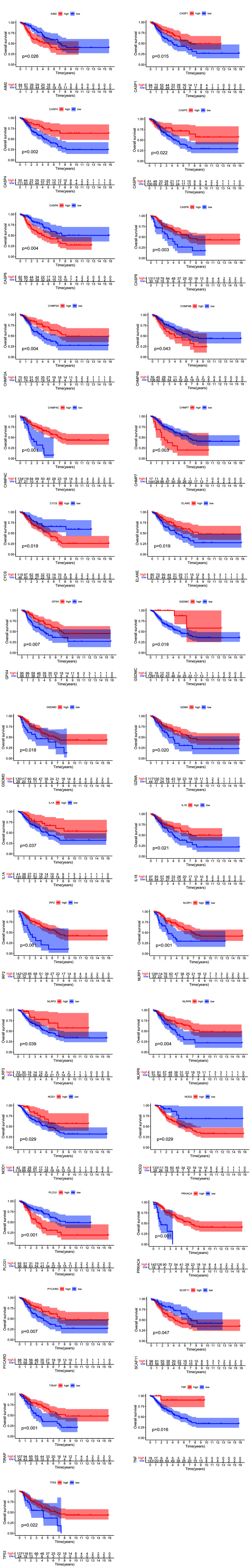


**Figure:**
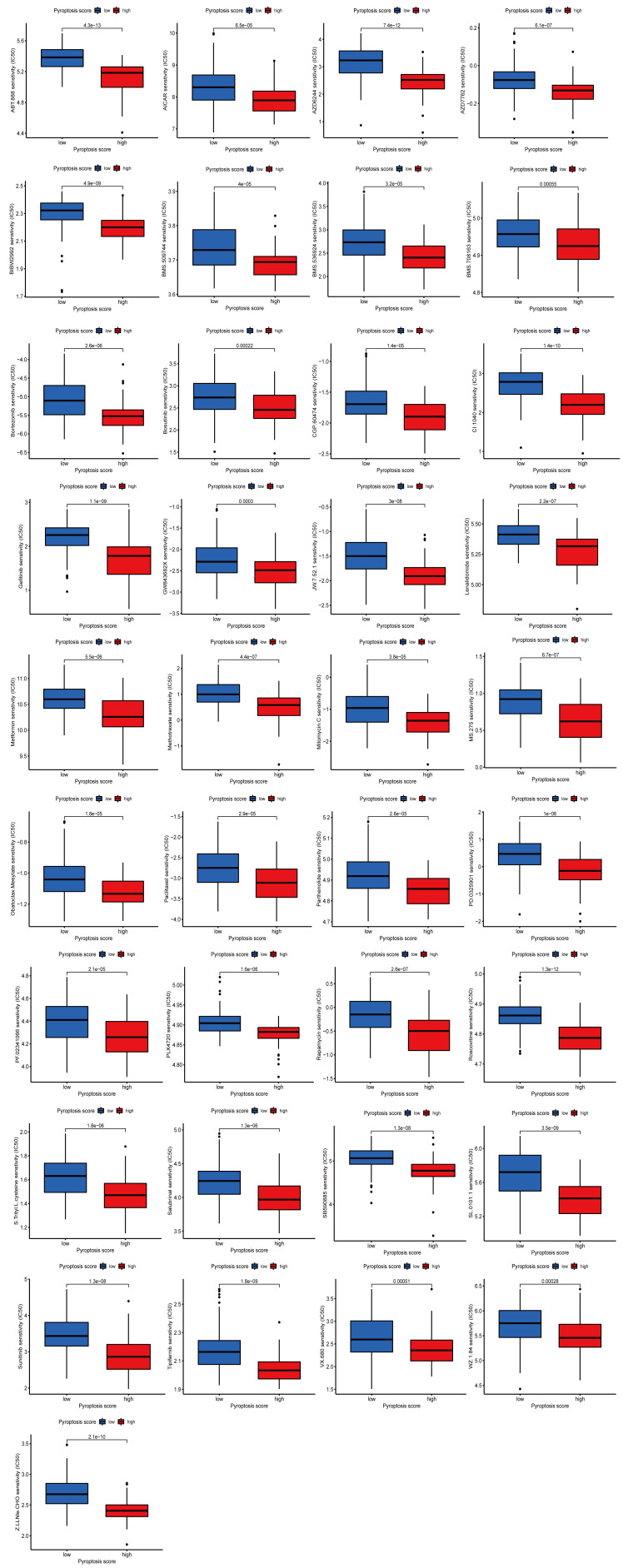


**Figure:**